# P-86. Utility of Metagenomic Next-Generation Sequencing in Detecting Pathogens Causing Orthopedic and ENT Infections

**DOI:** 10.1093/ofid/ofae631.293

**Published:** 2025-01-29

**Authors:** Alia McConnell, Anila Hussain, Tina B Edmonston, Monica Ianosi-Irimie, Dejan Nikolic

**Affiliations:** Cooper University Health Care, Camden, New Jersey; Cooper University Health Care, Camden, New Jersey; Cooper University Health Care, Camden, New Jersey; Cooper University Health Care, Camden, New Jersey; Cooper University Health Care, Camden, New Jersey

## Abstract

**Background:**

Traditional methods such as gram stain and cultures are recommended to identify pathogens from patient specimens. Metagenomic Next-Generation Sequencing (mNGS) is a new attractive technology to detect occult infections, that does not provide susceptibilities, with not well-established utility. We compared results of mNGS tests performed at Microgen Reference lab to those of traditional culturing on patient outcomes.

**Methods:**

This retrospective study includes all patients from Aug 2022 to Dec 2023 whose biopsy samples were submitted for both traditional culturing and mNGS diagnostic tests. Clinical and lab data were extracted from the electronic medical record. Patient outcomes when utilizing traditional versus mNGS diagnostic methods were compared.

**Results:**

53 orthopedics and ENT cases were studied. 31/41 (75.6%) orthopedic cases (spine, hips, and synovial fluid) had concordant results, with 19 (46.3%) concordant negative cases and 12 (29.3%) concordant positive cases showing similar organism(s). Of the discordant cases, 12.2% (5/41) mNGS results were accurate, and 9.75% (4/41) culture results were accurate. One specimen (2.44%) showed contamination with both methods. Of 12 ENT cases, overall concordance of accurate results was only 50%, 1 negative case and 5 cases showing the same organism(s) with both methods. 16.7% (2/12) the culture results were accurate while mNGS results were incorrect, and 4/12 (33.3%) specimens showed contamination by both methods.

**Conclusion:**

Utilizing mNGS versus traditional methods did not result in any changes in patient management like empirical and targeted antibiotic regimen or in hospitalization outcomes including duration of therapy or length of stay. The accuracy of the testing is equivalent, while mNGS is much more expensive and not readily available in clinical labs, resulting in longer TAT (6 days vs. cultures 3 days). The quality of results is mainly affected by the quality of the specimen (33% contamination for ENT specimens). These data ultimately guided the stewardship discussions about utilizing mNGS at CUHC to discourage mNGS testing for ENT sources unless the collection method is improved and reserve the utilization of mNGS for challenging orthopedic cases.

**Disclosures:**

**All Authors**: No reported disclosures

